# Deciphering the Properties and Functions of Glycoproteins Using Quantitative Proteomics

**DOI:** 10.1021/acs.jproteome.3c00015

**Published:** 2023-04-03

**Authors:** Senhan Xu, Xing Xu, Ronghu Wu

**Affiliations:** ^†^School of Chemistry and Biochemistry, and ^‡^the Petit Institute for Bioengineering and Bioscience, Georgia Institute of Technology, Atlanta, Georgia 30332, United States

**Keywords:** glycoproteomics, identification and quantification of glycoproteins, quantitative proteomics, mass spectrometry, protein glycosylation, SILAC, TMT

## Abstract

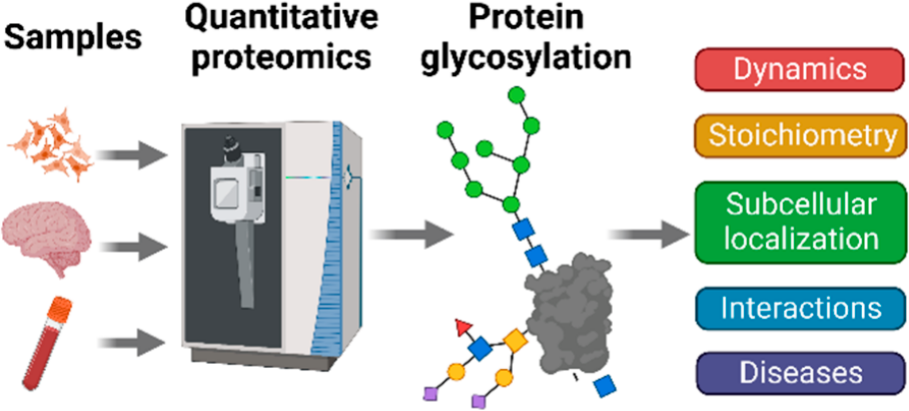

Glycosylation is one of the most common and important protein modifications, and it regulates the properties and functions of a wide range of proteins. Aberrant glycosylation is directly related to human diseases. Recently, with the advancement of mass spectrometry (MS) instrumentation and MS-based glycoproteomic methods, global characterization of glycoproteins in complex biological samples has become possible. Using quantitative proteomics, the abundance of glycoproteins in different samples can be quantified, which provides a wealth of information to further our understanding of protein functions, cellular activities, and the molecular mechanisms of diseases. In this review, we discuss quantitative proteomic methods used for comprehensive analysis of protein glycosylation, and cover the applications of quantitative glycoproteomics to unveil the properties and functions of glycoproteins and their association with various diseases. It is expected that quantitative proteomic methods will be extensively applied to explore the role of protein glycosylation in complex biological systems, and to identify glycoproteins as biomarkers for disease detection and as therapeutic targets for disease treatment.

## 1 Introduction

Protein glycosylation is ubiquitous in biological systems, and among 20 naturally occurring amino acids, the side chains of several of them in proteins (such as S, T, Y, C, K, R, N, and W) can be modified by glycans.^1−7^ Based on the prediction, about half of proteins in mammalian cells can be glycosylated.^8,9^ Protein glycosylation plays extremely important roles in cells, such as regulating protein folding and trafficking, and their interactions with other molecules. Aberrant glycosylation is directly related to human diseases including cancer and neurodegenerative diseases.^10,11^ There are two major types of protein glycosylation, i.e., *N-*glycosylation and mucin-type *O-*glycosylation. *N-*Glycosylation refers to glycans attached to the asparagine residue of proteins with a consensus sequence of *N-*X-S/T, where X can be any amino acid other than proline.^12^*N-*Glycans have the common GlcNAc_2_Man_3_ core structure with further elaborations in the ER and the Golgi apparatus.^13^ For mucin-type *O-*glycosylation, glycans are covalently attached to the serine and threonine residues starting with GalNAc. There are eight types of the core structures of mucin-type *O*-glycans, and the core 1 and core 2 glycans are most common in humans.^14,15^ Mucin-type *O-*glycans are typically smaller than *N-*glycans, but it may be more condensed on some proteins, such as mucins.^16^ A unique type of glycosylation, i.e., *O-*GlcNAcylation, mainly occurs in the nucleus and the cytoplasm. For this modification, *O-*GlcNAc is attached to the serine and threonine residues catalyzed by the sole enzyme of *O-*GlcNAc transferase (OGT), and is only removed by *O-*GlcNAcase (OGA).^17−19^ This modification is highly dynamic and is involved in crosstalk with other modifications, including phosphorylation.^19^ It was recently unveiled that this modification can occur cotranslationally, and it can modify many proteins in human cells.^20,21^

When a protein is glycosylated, its hydrophobicity, structure, dynamics, and interactions with other molecules can be dramatically changed. Despite the importance of protein glycosylation, it is still very challenging to globally and site-specifically analyze glycoproteins due to the following reasons. First, glycosylation is normally of low stoichiometry in cells and the abundance of parent proteins is often low. Therefore, it is critical to effectively enrich glycopeptides/glycoproteins prior to analysis.^22,23^ Second, the heterogeneity of glycan structures and the possible linkages of glycans to different amino acid residues of proteins make it difficult to unambiguously pinpoint the glycosylation sites and to deconvolute the glycan structures, which also makes the selective enrichment of glycopeptides/glycoproteins more challenging.^24,25^ With the development of selective enrichment methods and mass spectrometry (MS)-based proteomics, global analysis of protein glycosylation becomes possible.^26^ Currently, MS-based proteomics is very powerful to comprehensively analyze glycoproteins in complex biological samples.^27−31^ Coupling with quantitative methods, the abundance changes of glycoproteins in various samples can be measured, which greatly expedites the study of glycoprotein functions and the discovery of glycoproteins as potential biomarkers. In this review, we discuss the methods to systematically quantify glycoproteins using MS-based proteomics, including metabolic labeling-based methods, chemical labeling methods, label-free quantification, and enrichment and quantification using an isotopic-tagged cleavable linker, and their applications to study the properties and functions of glycoproteins in various samples and diseases.

## 2 Quantitative Glycoproteomic Methods for Glycoprotein Analysis

### 2.1 Metabolic Labeling-Based Methods

#### 2.1.1 Protein Labeling Using Heavy Amino Acids

Using stable-isotope labeling by amino acids in cell culture (SILAC), proteins can be labeled with heavy amino acids, allowing for their quantification by MS.^32^ Cells under different conditions or treatments are grown in the culture media supplemented with isotopically labeled amino acids. The heavy amino acids are incorporated into proteins in cultured cells. Different amino acids are used in SILAC experiments, and lysine and arginine are most commonly used because they are present ubiquitously on peptides generated from trypsin digestion. The incorporation of different compositions of heavy isotopes (lysine: K0, K4, and K8; arginine: R0, R4, R6, R10, and R17) can allow for labeling up to 15 samples for analysis simultaneously.^33^ This method has gained wide popularity in the proteomics community.^34^

SILAC has been extensively applied to study protein *N-*glycosylation. The dolichyl-diphosphooligosaccharide–protein glycosyltransferase subunits of STT3A and STT3B are the catalytic subunits of the oligosaccharyl transferase (OST) complex that are responsible for transferring the preassembled core N-glycan onto the asparagine residue of proteins.^35,36^ In order to investigate *N-*glycosylation sites that are dependent on STT3A or STT3B, the two genes were knocked out separately using CRISPR-Cas9, and the abundances of *N-*glycosylated proteins in each treatment were compared with those of the wild-type cells as a control.^37^ The N-glycans were deglycosylated before LC-MS/MS analysis. In total, 2190 glycosylation sites were identified from 892 proteins in HEK293 cells, and around 1000 *N-*glycosylation sites were quantified. The average abundances decreased more in the cells without STT3A than without STT3B, which is reasonable because STT3A is responsible for *N-*glycosylation on the translocon, while STT3B glycosylates some sites missed by STT3A.^38,39^ Further analysis revealed several new classes of STT3A-dependent acceptor sites, as well as a new class of STT3B-dependent sites that located in short loops of multispanning membrane proteins. To study the effect of *N-*glycosylation inhibition using tunicamycin on the protein secretion, the abundance changes of secreted proteins and glycoproteins were systematically quantified in yeast (*Saccharomyces cerevisiae*).^40^ Tunicamycin inhibits the synthesis of the core N-glycan, resulting in the inhibition of protein N-glycosylation. The results demonstrated the dramatic decrease of both proteins and glycoproteins in the secretome under the tunicamycin treatment, indicating the critical role of *N-*glycosylation in regulating protein secretion. On the other hand, a group of proteins were found to have a minimal abundance change under the tunicamycin treatment, suggesting that they might be secreted through nonclassical secretion pathways.^41,42^ With the treatment of tunicamycin in yeast cells, expectedly the abundances of many glycoproteins decreased dramatically, and around 5% of proteins were downregulated by more than 2-fold. These proteins are highly enriched in several glycan metabolism and glycolysis-related pathways.^43^

Besides protein *N-*glycosylation, SILAC has also been used for studying protein *O-*GlcNAcylation. Coupled with a chemoenzymatic enrichment method, it was found that the abundances of more than 10 *O-*GlcNAcylated proteins were markedly increased with the inhibition of glycogen synthase kinase-3 (GSK-3).^44^ Using a similar approach, a number of *O-*GlcNAcylated proteins were determined to be upregulated in cells responding to heat stress in Cos-7 cells.^45^ Combining SILAC with lectin weak affinity enrichment chromatography (LWAC) for enriching *O-*GlcNAcylated peptides, it was revealed that the *O-*GlcNAcylation level was altered in the cells with deficient polycomb repressive complex 2.^46^ Qin et al. employed SILAC together with pulse-chase labeling of *O-*GlcNAcylated proteins with a sugar analog containing an azido group that mimics endogenous *O-*GlcNAc. The dynamics of over 500 *O-*GlcNAcylated proteins were systematically quantified in NIH3T3 cells.^47^

**Figure 1:**
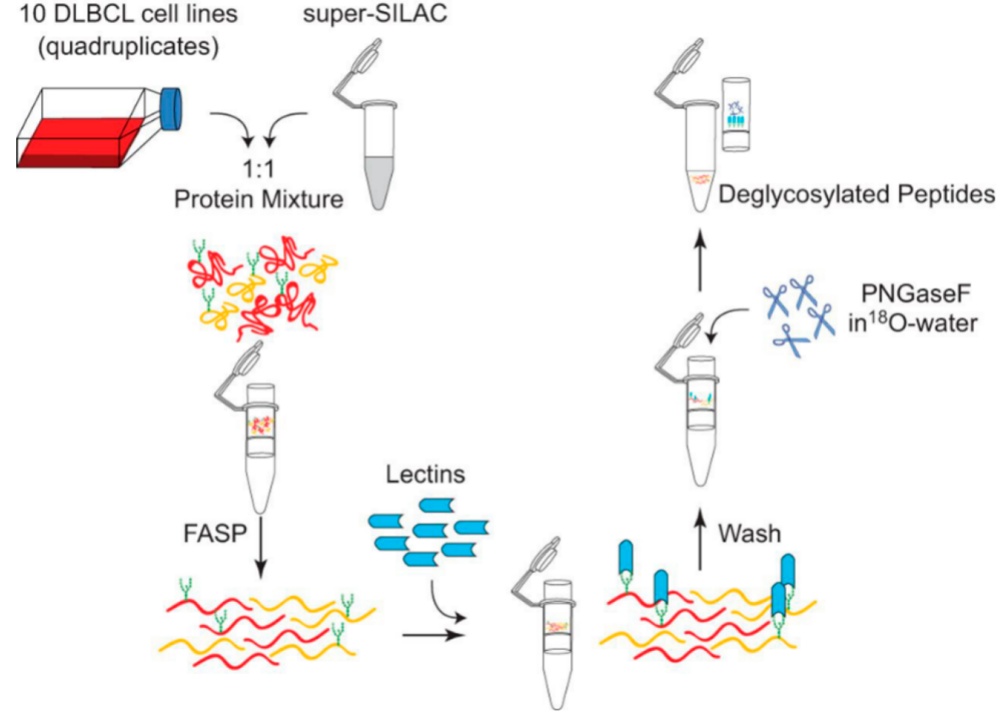
Workflow for lymphoma segregation using super-SILAC combined with *N-*glycopeptide enrichment. (Adapted from ref. (48), with the permission from Elsevier under the terms of the Creative Commons CC-BY license, which permits unrestricted use, distribution, and reproduction in any medium, provided the original work is properly cited.).

However, a major drawback of SILAC is that the metabolic labeling of proteins in the culture medium is not suitable for clinical samples, such as tissues and bodily fluids.^49,50^ To address this issue, a method termed super SILAC was developed (Figure 1).^51^ The samples from animals were spiked in with labeled proteins from different types of cells cultured in heavy media as a reference. Therefore, the abundance of glycoproteins in the tissue samples can be compared through their relative abundances against the corresponding heavy proteins from the cultured cells.^52^ Using this method, glycoproteins in the secretome of 11 cell lines were systematically quantified.^53^ Similarly, super-SILAC was used to quantify *N-*glycoproteins from patients with diffuse large B-cell lymphoma (DLBCL) to classify them into different lymphoma subtypes.^48^

#### 2.1.2 Glycan Labeling Using a Heavy Isotopic Sugar

**Figure 2:**
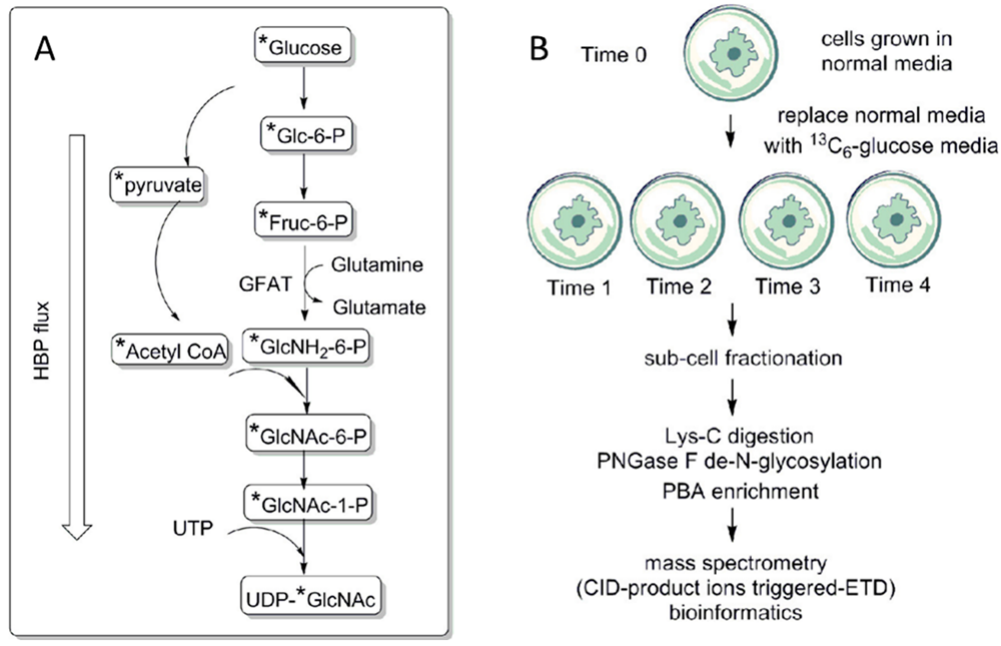
Quantification of the dynamics of *O-*GlcNAcylated proteins through metabolic labeling using ^13^C_6_-glucose. (A) Hexosamine biosynthetic pathway (HBP) and the metabolic labeling strategy of UDP-GlcNAc from ^13^C_6_-glucose. Heavily labeled species are marked with an asterisk. (B) Workflow for analysis of protein *O-*GlcNAcylation, including isotopic labeling, sample preparation, and mass spectrometric analysis. (Adapted and modified from ref. (55), with the permission from Elsevier under the terms of the Creative Commons CC-BY license, which permits unrestricted use, distribution, and reproduction in any medium, provided the original work is properly cited.).

Besides protein labeling using SILAC, it is also possible to label the glycan component for quantifying glycoproteins.^54^ For example, Wang et al. used ^13^C_6_-glucose to feed cells, which can be converted to ^13^C-labeled UDP-GlcNAc through the hexosamine biosynthetic pathway (HBP) (Figure 2).^55^ UDP-GlcNAc is used by OGT to modify glycoproteins. This metabolic labeling method was combined with boronic acid enrichment to determine the turnover rate of protein *O-*GlcNAcylation by quantifying *O-*GlcNAcylated proteins over time. O-GlcNAcylated proteins have overall slower degradation rates compared with phosphorylated proteins and acetylated proteins based on the previous quantification results.^56,57^

### 2.2 Chemical Labeling

#### 2.2.1 Tandem Mass Tag (TMT)

Because the heavy and light versions of a peptide will have different masses, MS spectra from a SILAC sample become more complex. This may affect the depth of protein analysis by MS, especially for data-dependent acquisition (DDA) that relies on the selection of most intense precursor ions. The oversequencing of peptides with high abundances (heavy and light versions of every peptide) may result in a decrease of total peptide identifications.^58^ Additionally, the incorporation of heavy amino acids is time-consuming, and the labeling efficiency may not be 100%, which could affect the quantification accuracy. An alternative method has become popular in recent years, in which peptides in different samples are chemically labeled with isobaric tags that are isotopically coded and have the same mass and chemical structure. When the samples are analyzed by MS, the same peptide from different samples has the exact same mass and elution time, so they are coselected for tandem MS analysis. In the tandem MS, the reporter ions are generated, and their intensities are used to accurately quantify the relative abundances of the peptide in different samples.^59^ One of the most common isobaric tag methods is called tandem mass tag (TMT). Based on the reactivity toward different functional groups (amine, thiol, and carbonyls), the reagents are classified into three types: TMT, IodoTMT, and AminoxyTMT. In general, TMT is very powerful for simultaneous analysis of multiple samples.^60^

**Figure 3:**
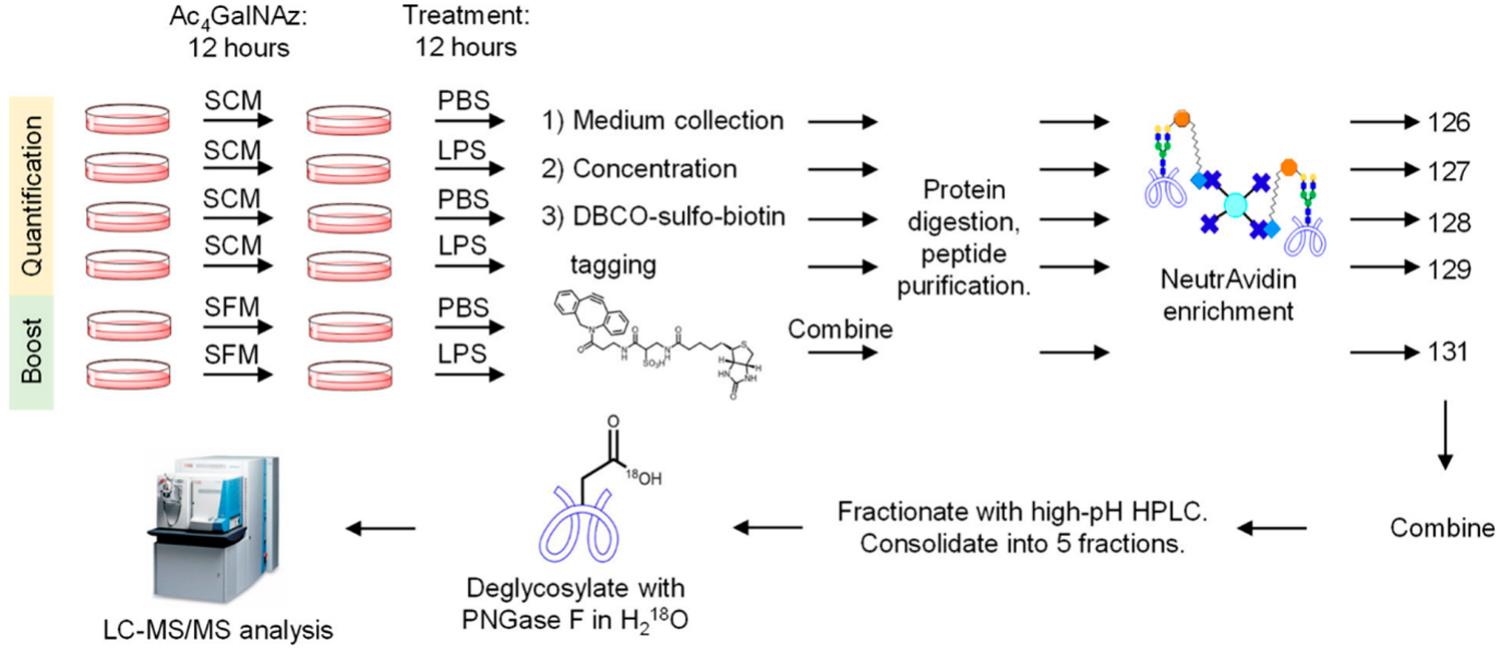
Experimental procedure for quantitative analysis of secreted glycoproteins coupling selective enrichment with a boosting approach. The sixplexed TMT reagents (channels 126, 127, 128, 129, and 131) are used to label the samples and the boosting one, respectively. (Adapted from ref. (61), with the permission from American Chemical Society.)

TMT was applied to quantify protein *N-*glycosylation in different samples. *N-*Glycan normally contains multiple monosaccharide units, and they could have different types of linkages, making the analysis of glycan structures difficult.^62^ A common practice to reduce the complexity of *N-*glycosylated peptides is the treatment with PNGase F, which can remove *N-*glycans if the core GlcNAc is not linked with an alpha 1,3-fucose.^63^ The deglycosylation process can be performed in heavy-oxygen water (H_2_^18^O), resulting in the generation of a small mass tag on *N-*glycosylation sites for MS analysis.^23,64,65^ The TMT labeling was coupled with *N-*glycoprotein enrichment to quantify the changes of secreted *N-*glycosylated proteins from monocytes and macrophages during the lipopolysaccharides (LPS) treatment (Figure 3). To enhance the detection of secreted glycoproteins with low abundance compared to highly abundant proteins in the serum, a boosting channel was added that contains enriched secreted *N-*glycoproteins from cells in serum-free media (SFM).^61^ Glycopeptides in the boosting channel were labeled together with the samples from serum containing media (SCM) using the TMT reagents, respectively, allowing for dramatic signal increase of glycopeptides at the MS1 and MS2 levels. In total, 308 unique glycopeptides were quantified from 178 glycoproteins. In contrast, without the boosting approach, only 103 unique glycopeptides were detected from 71 glycoproteins. Secreted glycoproteins upregulated during the LPS treatment were found to be associated with immune response and inflammation.

In another experiment, the abundance changes of surface glycoproteins from monocytes and macrophages treated with LPS were systematically quantified through combining metabolic labeling, bioorthogonal chemistry, and multiplexed proteomics.^66^ The time-resolved and site-specific responses of surface glycoproteins upon the LPS treatment and during the monocyte-to-macrophage differentiation were investigated. Differential remodeling of the surface glycoproteomes was observed among the cells, including the expression of new glycoproteins to the surface and the removal/internalization of existing surface glycoproteins. The surface glycoproteome changes in response to LPS between monocytes and macrophages showed some similarities and differences. Besides previously reported markers, novel surface glycoproteins quantified in the immune response process with dramatic alterations to the LPS treatment may serve as potential markers. Furthermore, site-specific protein glycosylation changes were found in different processes. For instance, glycosylation on N229 of granulocyte-macrophage colony stimulating factor receptor subunit alpha (CSF2RA) increased by 4.9 times in monocytes under the LPS treatment while the sites of N195 and N223 remained relatively similar. This work results in a better understanding of the important roles of cell-surface glycoproteins in the immune response during bacterial infection and potentially the identification of surface glycoproteins as disease biomarkers and drug targets.

**Figure 4:**
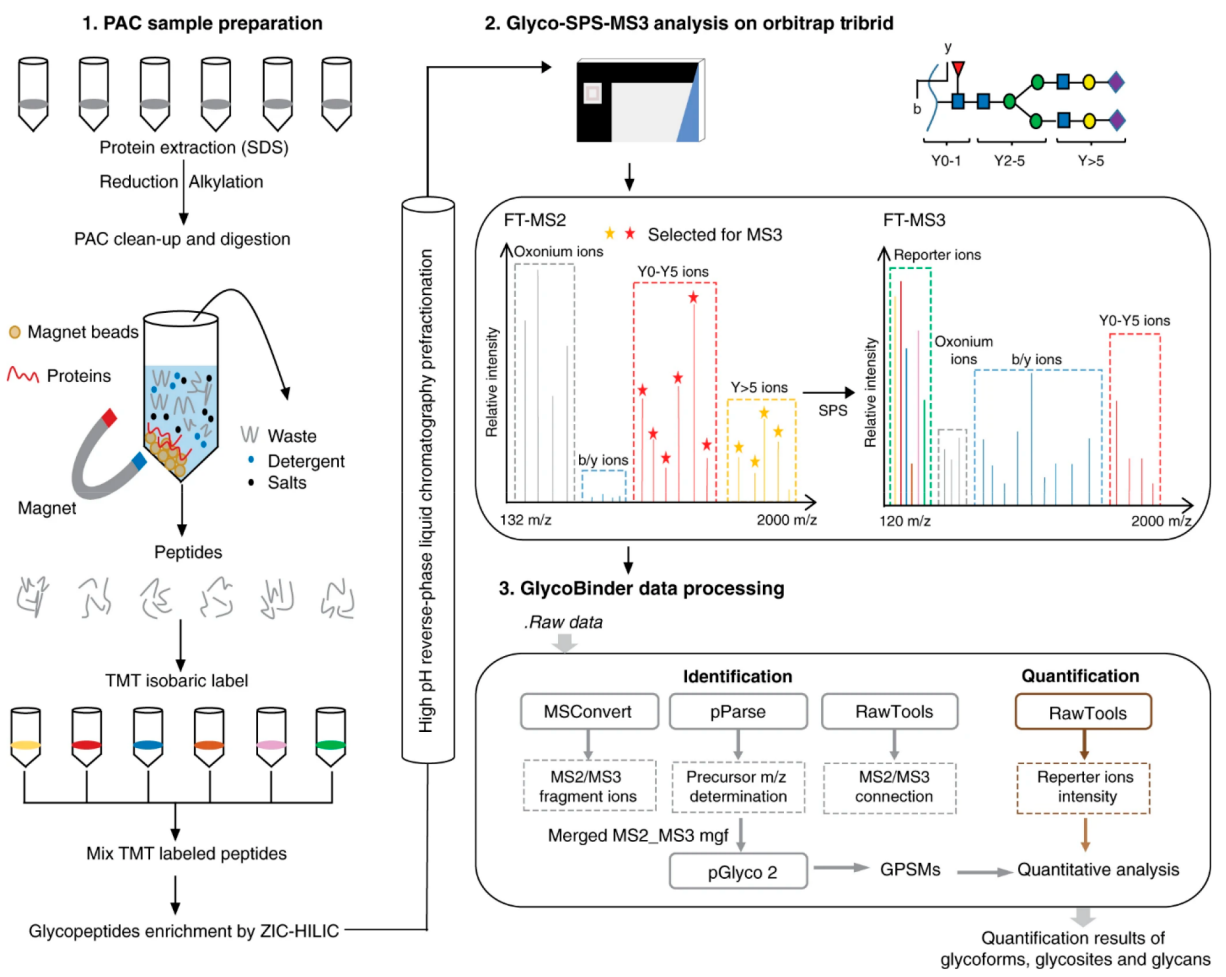
Workflow of SugarQuant. SugarQuant comprises (i) lysis of cells in the presence of SDS, (ii) protein extraction, (iii) protein concentration and endoproteolytic digestion using protein aggregation capture (PAC), (iv) multiplex TMT labeling, (v) *N*-glycopeptide enrichment by zwitterionic HILIC (ZIC-HILIC), followed by (vi) basic reverse phase (bRP) prefractionation, and (vii) LC-MS3 analysis using Glyco-SPS-MS3, which generates high-resolution MS2 and MS3 product ion scans. Data processing using the GlycoBinder tool (viii) combines MS2 and MS3 fragment ions for *N*-glycopeptide identification, and extracts TMT reporter-ion intensities from MS3 scans for each identified *N*-glycopeptide-spectrum-match (GPSM). (Adapted from ref. (71), which is under Creative Commons Attribution 4.0 license that permits use, sharing, adaptation, distribution and reproduction in any medium or format.)

Due to the heterogeneity of glycans and their frangibility during MS analysis, the analysis of deglycosylated peptides can help achieve higher glycoproteome coverage.^67−69^ However, with the development of MS instruments and searching software, characterization of TMT-labeled glycopeptides has become attractive because besides glycosylation sites, the glycan structure and composition information can be obtained.^70^ Fang et al. developed a pipeline for quantification of intact *N-*glycopeptides (Figure 4).^71^ As the optimal dissociation energies are different for glycan and peptide backbone fragmentation, the TMT-labeled glycopeptides were first fragmented using high-energy collision dissociation (HCD) with lower (25%) normalized collision energy (NCE) and the fragments were recorded in the Orbitrap. The ten most intense ions in MS2 generated by HCD in the high mass range were coselected for further dissociation with higher energy (35%–40%) and the resulting ions were detected. This setup enabled analysis of complex *N-*glycans using their glycan fragments in MS2, and accurate quantification of the TMT reporter ions and identification of the peptide backbone were performed in MS3. The method termed SugarQuant was applied for quantification of *N-*glycoproteins in 2FF-treated human Burkitt’s lymphoma cells. In another study, the abundance changes of glycoproteins in mouse embryonic stem cells (mESC) were quantified with α-1,3-fucosyltransferase Fut9 or the fucose transporter Slc35c1 knocked out.^72^ Both Fut9 and Slc35c1 knockout (KO) cell lines were ricin resistant. Deletion of Slc35c1 abolished fucosylation of *N-* and *O-*glycoproteins, and the glycans affected by the loss of Fut9 were a subset of those targeted by Slc35c1. Among the glycoproteins affected by both mutations, six of them involving in the process of ricin toxicity were validated.

TMT was also applied to quantify *O-*GlcNAcylated proteins in brain samples from patients with Alzheimer’s disease. The abundances of some *O-*GlcNAcylated proteins were altered in Alzheimer’s disease, including ankyrin-G (ANK3), synaptopodin (SYNPO), and A-kinase anchor protein 11 (AKAP11), which could serve as potential biomarkers for disease detection.^73^ Protein *O-*GlcNAcylation is the only known type of glycosylation existing in the nucleus of mammalian cells.^74^ To quantify the nuclear-cytoplasmic distribution of *O-*GlcNAcylated proteins, glycopeptides enriched from the nucleus and the cytoplasm were labeled using TMT.^75^ It was found that *O-*GlcNAcylated proteins with different functions have distinct distributions.

#### 2.2.2 Other Chemical Labeling-Based Methods

Besides TMT, another isobaric labeling method, i.e., isobaric tags for relative and absolute quantitation (iTRAQ), was also applied for glycoproteomic analysis. Similar to TMT, it consists of a reporter ion, a balance group, and an amine reactive group (NHS) that targets the *N-*termini and the lysine residues of peptides.^76,77^ For example, Shi et al. labeled glycopeptides in the samples from patients with Alzheimer’s disease and the controls using iTRAQ.^78^ This method was also employed to compare glycoproteins from tears, the extracellular fluid of epithelial cells covering the surface of the eye from patients with climatic droplet keratopathy (CDK).^79^ Both TMT and iTRAQ are commercially available, but are expensive. A more cost-effective alternative i.e., DiLeu, was developed in Dr. Li’s lab.^80^ The reagent only takes one or two steps to synthesize, and the material cost for a labeling experiment is less than $5 for 100 μg of protein digest per channel.^81^ In contrast, a set of TMT sixplex (0.8 mg for each channel) reagents costs more than $700. With further improvement, it can label up to 21 different samples with its newest generation.^82,83^ Using the 12-plexed DiLeu reagents, Wang et al. employed a boosting approach to analyze glycoproteins from the cerebrospinal fluids from patients with Alzheimer’s disease.^83^

### 2.3 Label-Free Quantification (LFQ)

**Figure 5:**
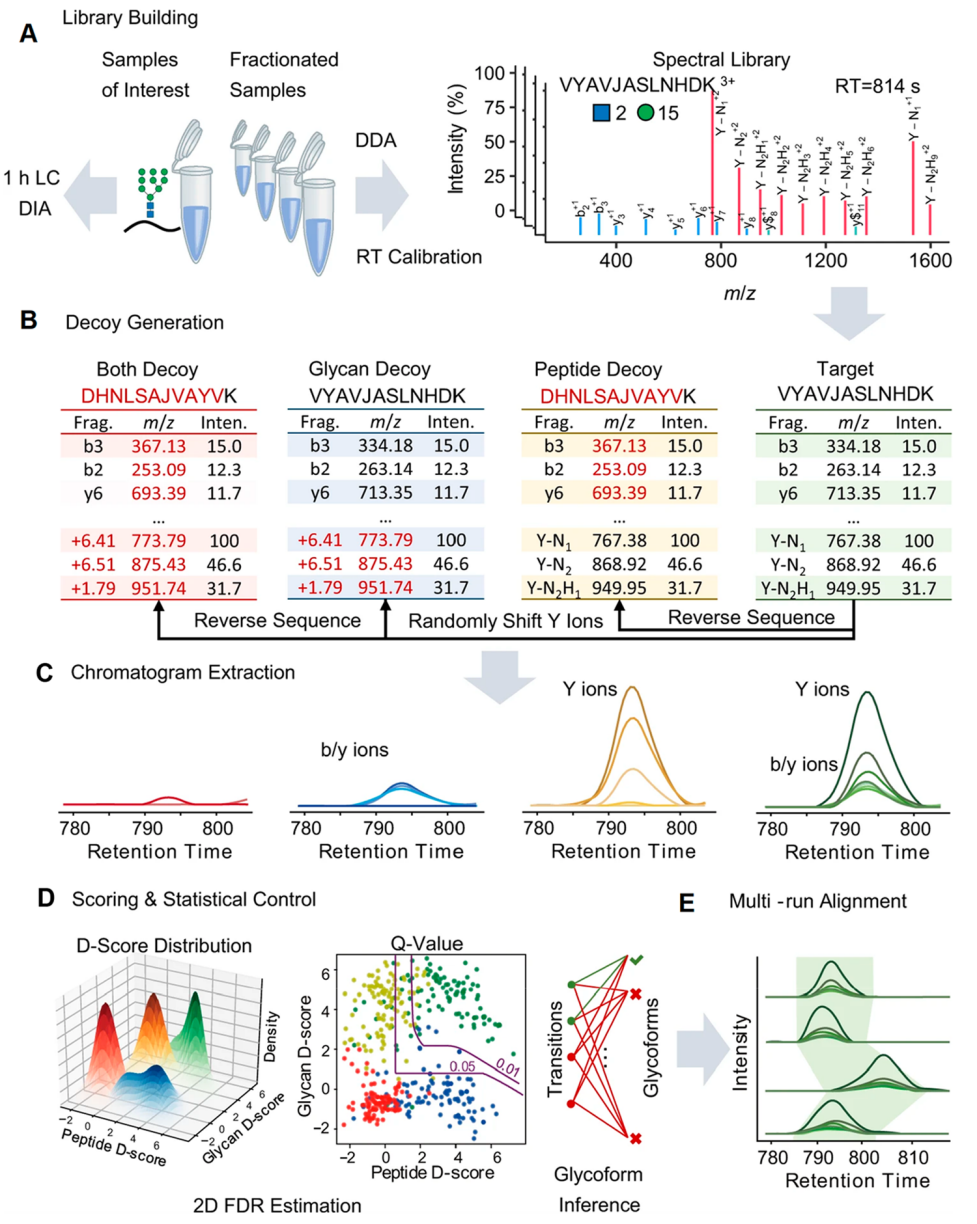
Workflow of GproDIA. (A) Building a spectral library of glycopeptides containing peptide ions (blue lines for b/y, and green lines for b/y with one HexNAc or its cross-ring fragment) and glycan Y ions (red lines) by DDA. “J” in peptide sequence indicates the *N-*glycosylation site. The glycan symbols are as follows: a green circle or “H” represents Hex; a blue square or “N” represents HexNAc. (B) Generating peptide decoys, glycan decoys and both decoys. (C) Extracting chromatogram features of the target glycopeptides and the decoys from the DIA data. (D) Scoring the extracted features and estimating error rates by a 2-dimentional FDR approach and a glycoform inference strategy. (E) Performing multirun alignment to reduce missing values. (C–E) Green color indicates target peak groups, yellow indicates peptide decoy peak groups, blue indicates glycan decoy peak groups, and red indicates both decoy peak groups. (Adapted from ref. (86), which is under Creative Commons Attribution 4.0 license that permits use, sharing, adaptation, distribution, and reproduction in any medium or format.)

Compared with labeling methods, label-free quantification does not require the chemical or metabolic labeling of samples and can compare the abundances of the same peptides from different samples based on their intensities at the MS1 level. This is particularly beneficial when the sample amount is very limited, or speedy handling of the samples is required, or the number of samples is too large. Glycoproteomic profiling with LFQ revealed the change of *N-*glycosylation signature in pancreatic ductal adenocarcinoma, and *N-*glycosylation increased on glycoproteins such as apolipoprotein B-100 (APOB) and biglycan (BGN), and in several cancer associated pathways (TGF-β, TNF, NF-κB, and TFEB-related lysosomal changes).^84^ Using a similar approach, the expressions of *N-*glycoproteins in different infection stages of *M. oryzae* were quantified.^85^ In recent years, using data independent acquisition (DIA) for intact glycopeptide analysis has become popular due to the improved scanning speed and sensitivity of MS. Dong et al. constructed an intact glycopeptide library based on the MS2 spectra from data dependent acquisition (DDA) and used it for spectral matching in the DIA analysis. The Y-type ions from glycans were used for accurate quantification.^27^ Later, Yang et al. developed a platform called GproDIA, which can accurately identify and quantify glycopeptides using DIA with a 2-dimensional false discovery rate (Figure 5). The framework enables the accurate characterization of intact glycopeptides using wide isolation windows.^86^ LFQ was also applied to site-specifically quantify protein *O-*GlcNAcylation changes during the T cell activation.^87^ However, the precision of LFQ is relatively lower compared with metabolic and chemical labeling methods because when the same peptides in different samples are measured, deviations may be caused during sample preparation and under different machine conditions.

### 2.4 Enrichment and Quantification Using an Isotopic-Tagged Cleavable Linker

**Figure 6:**
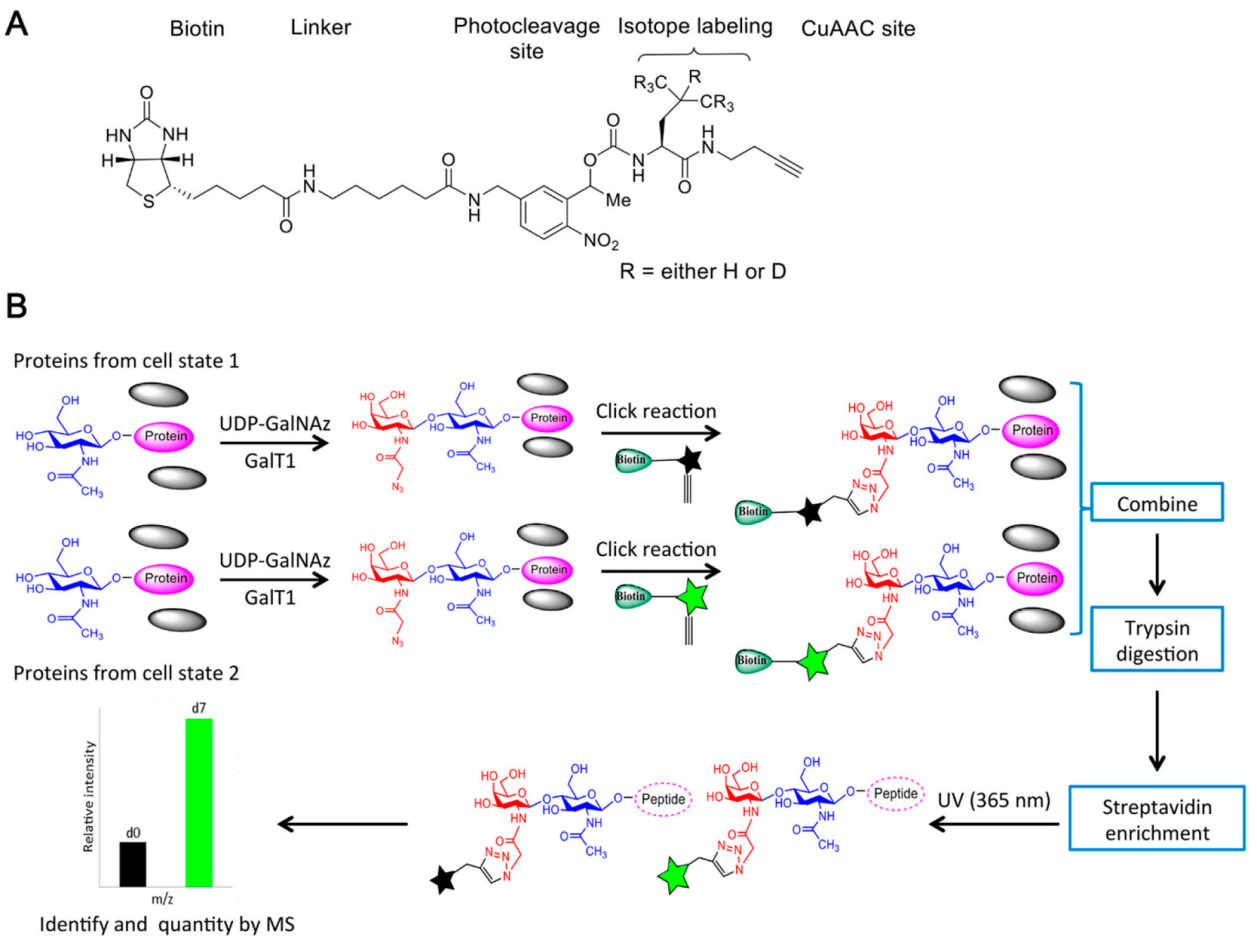
(A) Chemical structure of the isotope labeled cleavable linker for glycoprotein enrichment. (B) The workflow of glycopeptide enrichment and quantification. (Adapted from ref. (90), with permission from American Chemical Society.)

Most glycoproteins in cells are of low abundance, and thus glycopeptide/glycoprotein enrichment is imperative for their global analysis. This process can be laborious and technically challenging due to the heterogeneity of glycans. To enrich glycopeptides, a tag can be added specifically to glycans through chemical or enzymatic reactions. The tag can be isotopically encoded to facilitate glycopeptide identification or quantification by MS. For example, elements with a unique isotopic signature and that rarely occur in the proteome (such as bromine) were incorporated to the cleavable linker, and the distinctive precursor peak patterns in MS1 can be exclusively recognized and selected by MS for further fragmentation, which increases the specificity of MS analysis because nonglycopeptides were excluded.^87,88^ Additionally, once the tag is isotopically labeled, glycopeptides from various samples can be distinguished due to the distinctive masses encoded on the tags. Qin et al. labeled *O-*GlcNAcylated proteins using a chemoenzymatic method that added GalNAz to *O-*GlcNAc (Figure 6). The azido group of GalNAz can react with the acid cleavable linker with a biotin moiety for selective enrichment. The linker contains isotopically coded elements that retain on glycopeptides after enrichment and cleavage, allowing for the quantification of two different samples based on their intensities on MS1, of which the peaks resembles those from SILAC.^89^ The authors applied the method to compare the stoichiometry of more than 100 *O*-GlcNAcylation sites between the placenta samples from male and female mice. It was found that the overall *O*-GlcNAcylation stoichiometry is higher in the female placentae than the male ones. Similarly, Li et al. developed an isotope-coded UV cleavable linker for quantitative profiling of protein *O-*GlcNAcylation.^90^

## 3 Quantitative Glycoproteomics for Studying the Properties and Functions of Glycoproteins

### 3.1 Systematic Investigation of the Dynamics of Glycoproteins

In cells, proteins are actively degraded and synthesized, which is directly related to cell survival and cellular activities.^91^ Glycosylation can have a profound impact on protein dynamics by regulating protein folding and degradation.^92,93^ Previously, it was found that *O-*GlcNAcylation can affect the dynamics of many proteins to regulate their functions. For example, Myc proto-oncogene protein (c-MYC) is a critical transcription factor in cancer progression. It was reported that c-MYC can be *O-*GlcNAcylated, and the modification stabilized the protein in prostate cancer cells.^94,95^ Moreover, *O-*GlcNAcylation promoted the trafficking of NOTCH1 to the cell surface and mediated its stability.^96^

**Figure 7:**
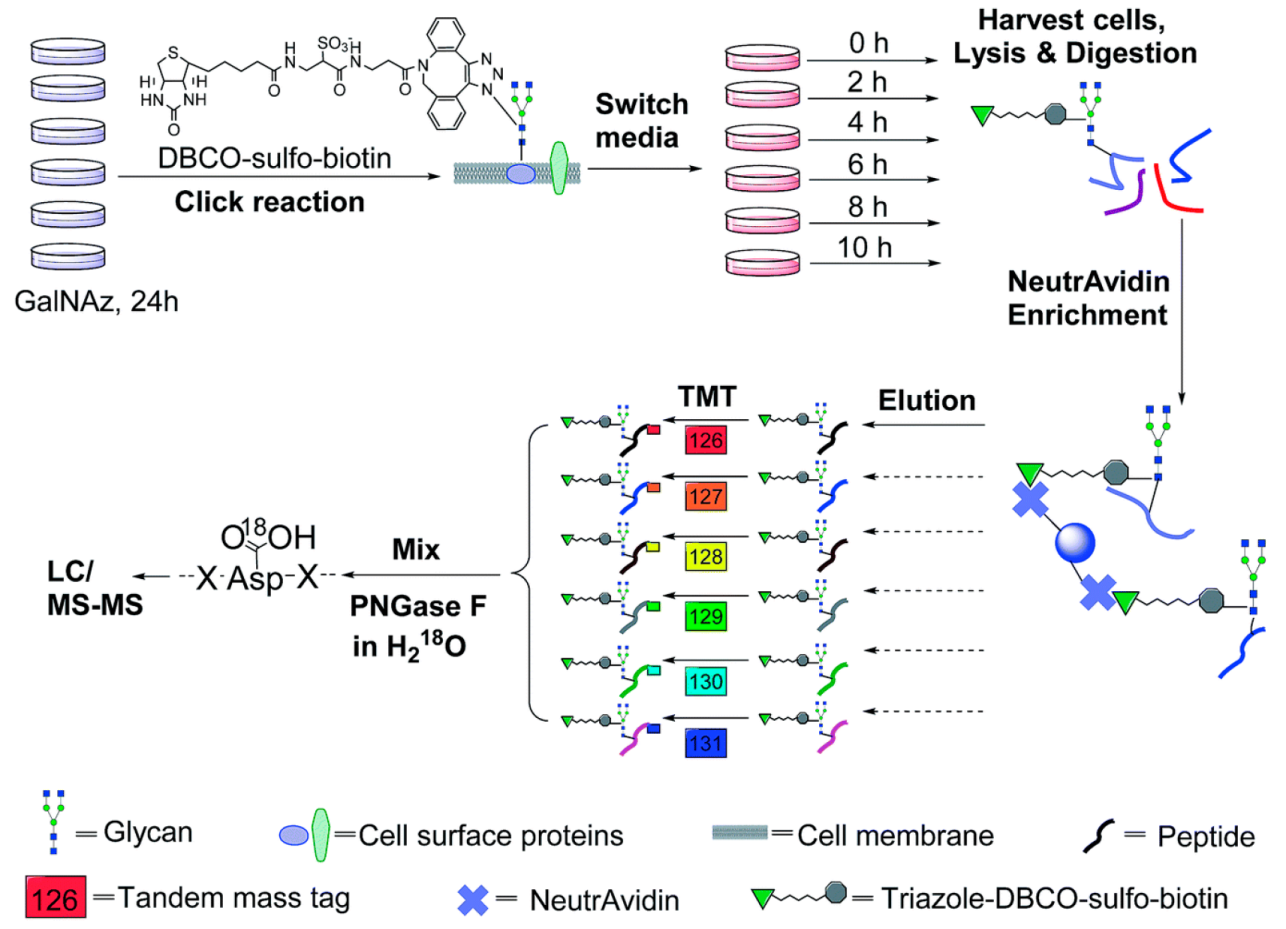
Experimental procedure for studying surface glycoprotein dynamics and measuring their half-lives using pulse-chase SILAC coupled with TMT. (Adapted from ref. (103), with the permission from Royal Society of Chemistry.)

Besides single proteins, it is of great value to study the effect of glycosylation modulating protein stability on a global scale. The most popular method to study protein dynamics is pulse-chase SILAC, in which the cell culture medium is switched from heavy to light or from light to heavy initially, and the abundance changes of the existing proteins labeled with heavy or light amino acids are quantified over time.^97,98^ This method was also successfully applied to study the dynamics of glycoproteins.^99,100^ However, using SILAC alone may not have enough time points for accurate quantification of the abundance changes of proteins over time. To overcome this issue, a method coupling pulse-chase SILAC with TMT was introduced, taking advantage of the power of TMT in sample multiplexing.^101,102^ For example, Xiao et al. integrated pulse-chase SILAC, selective enrichment of surface glycoproteins and TMT-based multiplex proteomics to study the degradation of cell-surface glycoproteins (Figure 7).^103^ The experimental results demonstrated that surface glycoproteins with catalytic activities were more stable than those with binding or receptor activities. Cell-surface proteins are exposed to different environments, but glycans on surface proteins may provide one layer of protection, especially for proteins with catalytic activity. Additionally, as proteins were actively synthesized in the chase period, the accumulation of glycoproteins with heavy amino acids provided an opportunity to study the synthesis of glycoproteins over time. Therefore, the pulse-chase labeling enables the quantification of both the degradation and synthesis rates of surface glycoproteins simultaneously.^104^

Using the pulse-chase SILAC-TMT method, the degradations of *O-*GlcNAcylated proteins in the nucleus and the cytoplasm were quantified.^75^ It was revealed that the degradation of *O-*GlcNAcylated proteins between the nucleus and the cytoplasm was markedly different. Furthermore, the degradation rate of *O-*GlcNAcylated proteins and their corresponding nonmodified form were compared, and it was found that most proteins were stabilized by *O-*GlcNAcylation in both the nucleus and the cytoplasm. Besides protein degradation, the pulse-chase SILAC-TMT method can also be used to study the protein abundance change in response to stimuli. For example, the surface and secreted glycoprotein dynamics in response to LPS treatment in immune cells was systematically quantified.^61,66^ In another study, the change of surface glycoproteins during macrophage differentiation was studied using SILAC-TMT.^105^

### 3.2 Measurement of the Stoichiometry of Protein Glycosylation

Quantitative proteomics was applied to determine the stoichiometry of protein glycosylation. A method was developed for global analysis of the *N-*glycosylation stoichiometry, and the stoichiometries of over 100 *N-*glycoproteins were determined in a human ovarian cancer cell line (OVCAR-3).^106^ Alternatively, Xu et al. used Endo-H to treat glycopeptides, which left a GlcNAc residue linked to the asparagine side chains of peptides. The intensities of glycopeptides were compared with the corresponding nonmodified peptides in sequential window acquisition of all theoretical mass spectra (SWATH-MS) analysis to calculate the stoichiometry.^107^ Similarly, Yang et al. quantified the *N-*glycosylation stoichiometry by comparing the intensities of PNGase F-treated *N-*glycopeptides with the corresponding nonmodified peptides.^108^ However, the ionization efficiencies of the glycosylated (PNGase F-treated) and nonglycosylated forms of a peptide may be different.

To quantify the stoichiometry of a specific *O-*GlcNAcylated protein, a gel-based method using chemoenzymatic labeling of *O-*GlcNAcylated proteins was developed.^109^ In this method, the *O-*GlcNAcylated proteins were labeled with a large mass tag that was over 10k Da, and the mass difference allowed for separation of the glycosylated and nonglycosylated forms of a protein on gel. The stoichiometry of glycoproteins was determined by comparing the signal intensity of the glycoprotein with that of the nonmodified form using Western blotting. However, the method cannot be applied to globally study the stoichiometry of protein *O-*GlcNAcylation because the mass tag is too large that is not suitable for MS analysis.

### 3.3 The Localization of Glycoproteins in Different Subcellular Compartments

The subcellular localization of proteins is closely related to their functions. For example, cell-surface glycoproteins play vital roles in regulating cell–cell communication and recognition of signaling molecules and pathogens. Wollscheid et al. developed an elegant method called cell surface capture (CSC) for interrogating cell-surface glycoproteins using MS-based proteomics.^110^ The method was extensively applied to study surface glycoproteins, including the quantification of differential expression of surface glycoproteins in different types of cells and in various processes.^111−116^

Metabolic labeling with a sugar analog is very powerful to capture cell-surface glycoproteins.^117,118^ The azido/alkynyl group of labeled glycoproteins allows for specific enrichment of extracellular glycoproteins.^117,119−121^ Coupling with multiplexed proteomics, the method was applied to quantify cell-surface glycoproteins in human glioblastoma tumors and statin-treated liver cells.^122,123^ Recently, with the development of proximity labeling strategy, cell-surface proteins can be biotinylated using genetically encoding horseradish peroxidase (HRP) and the supplement of biotin-phenol in the media.^124^ Quantitative analysis of cell-surface proteins in olfactory projection neurons of *Drosophila* revealed the downregulation of wiring molecules and the upregulation of synaptic molecules in the transition from developing to mature projection neurons. It is possible that the proximity labeling strategy can be expanded to study glycoproteins in the ER and the Golgi apparatus.

Many secreted proteins are glycosylated, and their glycosylation may change in cells under different conditions. Witzke et al. quantified the change of secreted glycoproteins during T cell activation using LFQ, and found that the abundances of 59 glycoproteins changed significantly during the T cell activation compared to the inactive state.^125^ Tüshaus et al. developed a miniaturized secretome analysis method termed “high-performance secretome protein enrichment with click sugars” (hiSPECS). In couple with LFQ, the method enabled the quantification of secreted proteins from primary astrocytes, microglia, neurons, and oligodendrocytes with the LPS treatment. Additionally, it identified secreted proteins resulting from the proteolytic cleavage of surface proteins by the Alzheimer-linked protease BACE1.^126^

**Figure 8:**
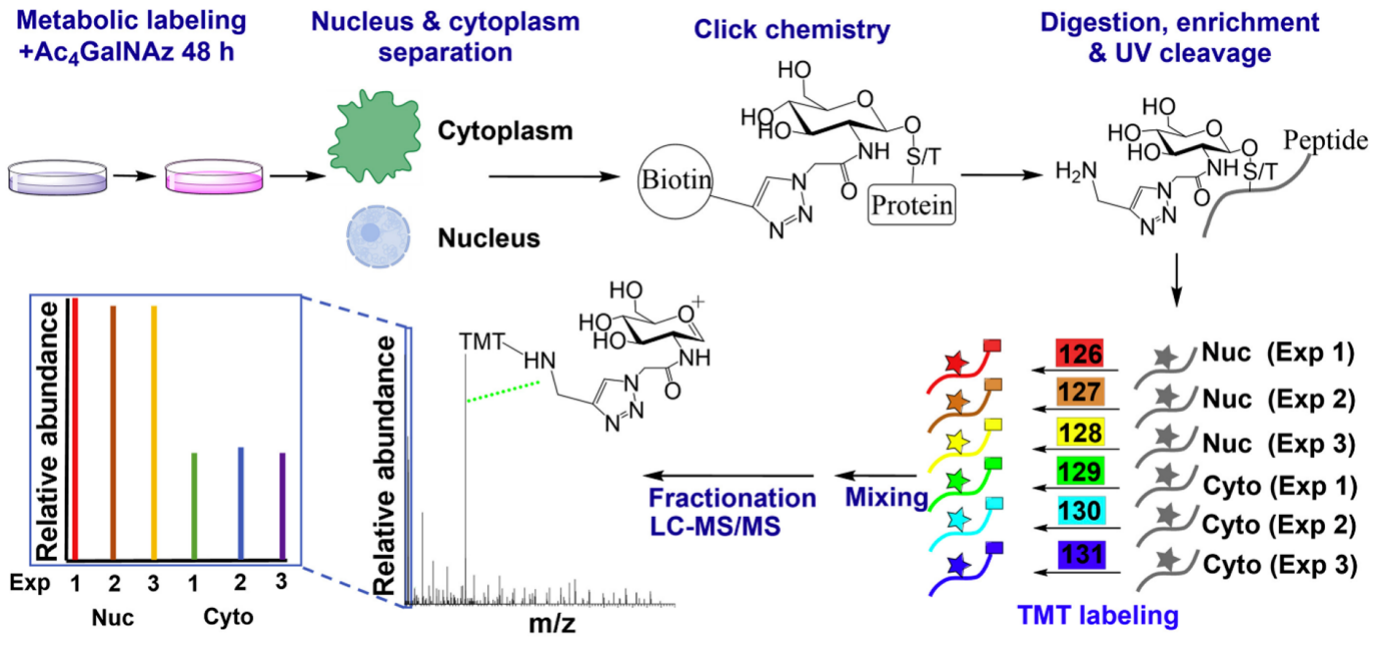
Quantification of the distributions of *O-*GlcNAcylated proteins in the nucleus and the cytoplasm. (Adapted from ref. (75), with the permission from Elsevier.)

For *O-*GlcNAcylation, it primarily modifies proteins in the nucleus and the cytoplasm. For the first time, we have systematically quantified the nuclear-cytoplasmic distribution of *O-*GlcNAcylated proteins (Figure 8).^75^*O-*GlcNAcylated proteins with different functions have distinct distributions. Furthermore, unique *O-*GlcNAcylation sites identified from the same protein can have different distributions. Some mitochondrial proteins were reported to be *O-*GlcNAcylated,^127,128^ and *O-*GlcNAcylated proteins in the mitochondrion had bidirectional changes in diabetes.^129^ Recently, it was found that *O-*GlcNAcylation modified proteins in the centrosome, the lysosome, and on the cell surface can regulate cell cycle, cancer progression, and cell–matrix interactions.^130−133^ It is of great interest to use glycoproteomic strategies to systematically identify and quantify protein *O-*GlcNAcylation in these cellular compartments. Despite the technical difficulties to isolate these organelles, the proximity labeling methods, such as APEX, TurboID, and BioID, could accelerate the investigation of compartment-specific *O-*GlcNAcylation.^134−136^

### 3.4 The Regulation of Protein Interactions with Macromolecules by Glycosylation

**Figure 9:**
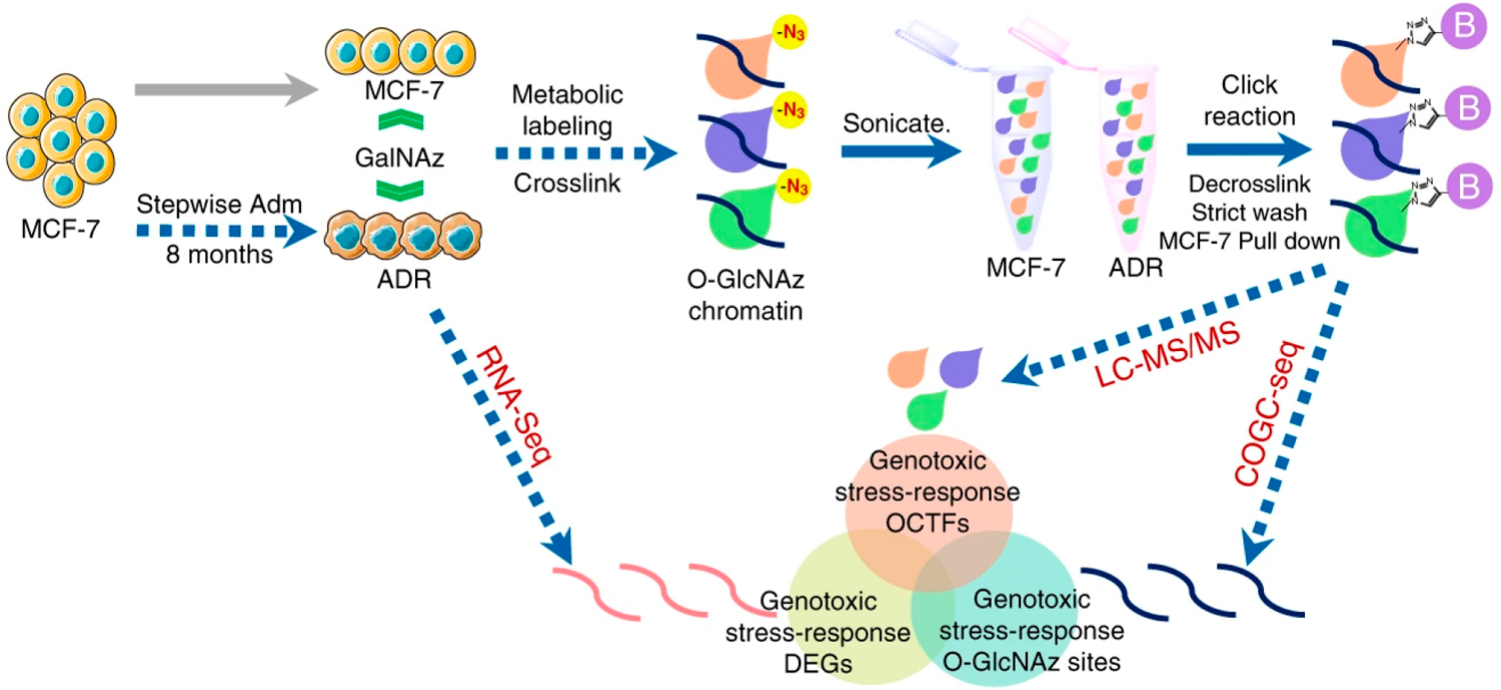
Schematic of the multiomics strategy involving a genome-wide chemical reporter-based method to study change of chromatin associated *O-*GlcNAcylated proteins during genomic stress. (Adapted from ref. (140), with permission from American Chemical Society.)

Glycosylation can result in the change of protein structure, solubility, stability, and interactions with DNA/RNA/proteins/other molecules. King et al. studied the thermal stability of proteins modulated by *O-*GlcNAcylation, and they found 72 proteins exhibiting *O-*GlcNAcylation-dependent thermostability changes.^137^ The thermostability changes related to *O-*GlcNAcylation could be due to different factors, including the alteration of protein structures by glycosylation, or the changes of the interactions with other molecules in cells facilitated/inhibited by *O-*GlcNAcylation. *O-*GlcNAcylation modifies a large number of proteins involved in transcription. To study *O-*GlcNAcylated proteins associated with transcription and chromatin binding, the chromatin was isolated from the nucleus, and *O-*GlcNAcylated proteins were enriched and identified (Figure 9).^138,139^ Under genotoxic stress, *O-*GlcNAcylated protein interactions with the chromatin were enhanced.^140^ Meanwhile, the enrichment of *O-*GlcNAcylated proteins interacting with the chromatin enabled the simultaneous enrichment of DNAs binding with glycoproteins. In a pulse-chase experiment, the DNA abundance change over time measured by next-generation sequencing revealed the turnover rate of *O-*GlcNAcylated proteins associated with the chromatin.^141^*O-*GlcNAcylation is also critical in regulating membraneless organelles, such as stress granules (SG), and liquid–liquid phase separation (LLPS). For example, *O-*GlcNAcylation is required for the formation of SG.^142^ On the other hand, *O-*GlcNAcylation is a suppressor of LLPS for the SynGAP/PSD-95 complex.^143^

**Figure 10:**
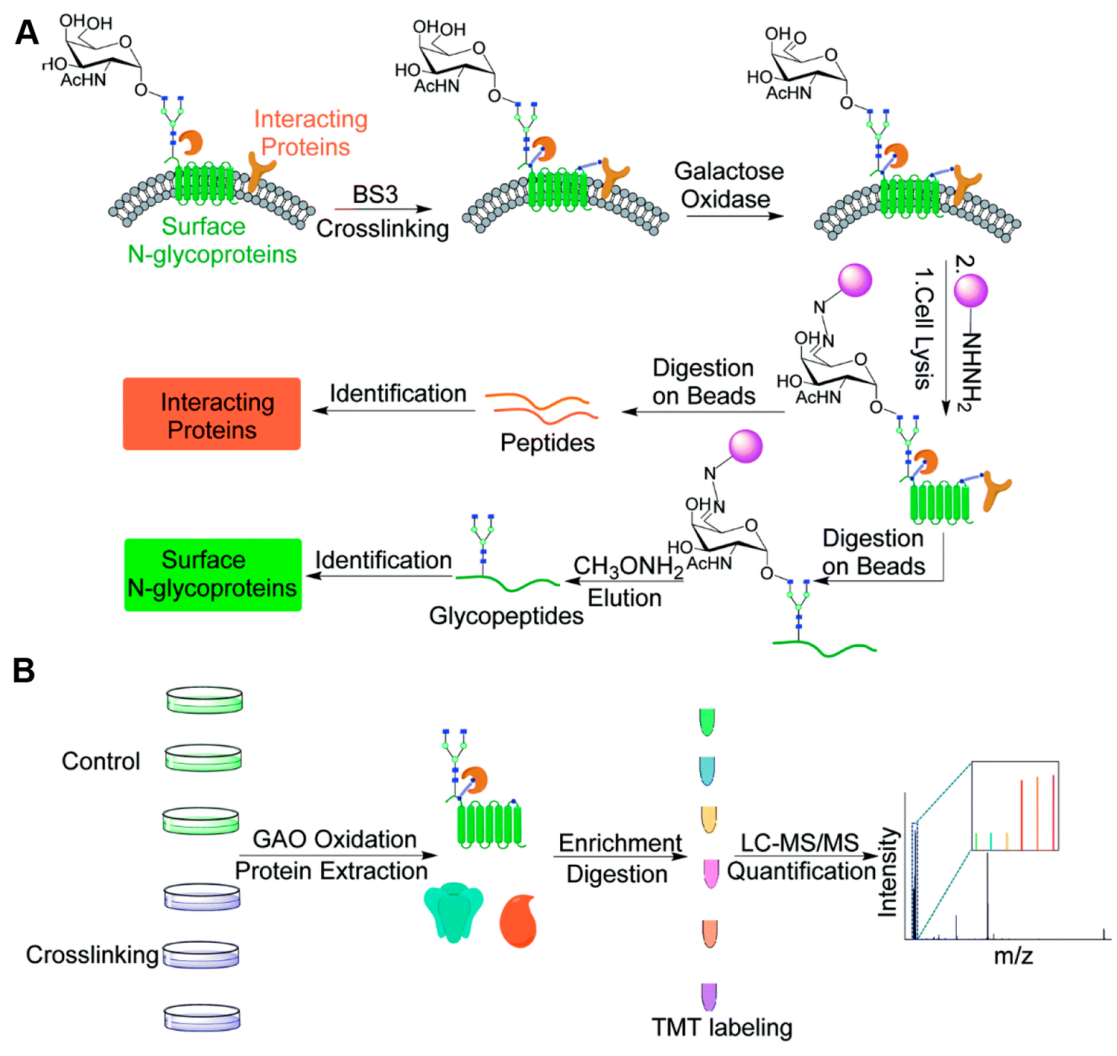
(A) Experimental procedure for investigating the cell surface glycoprotein interactions by integrating chemical cross-linking, an enzymatic reaction, and MS-based proteomics. (B) Detailed procedure for identification and quantification of proteins interacting with surface glycoproteins using multiplexed MS-based proteomics. (Adapted from ref. (146), with permission from Royal Society of Chemistry.)

*O-*GlcNAcylation can modulate protein–protein interactions. To globally identify the binding partners of *O-*GlcNAcylated proteins, a chemical reporter that contains a diazirine at C2 position can covalently link to its binding targets under the UV radiation.^144^ The similar strategy was applied to profile *N-*glycoproteins that interact with exogenous glycan-binding proteins (galectin-1 and cholera toxin subunit B) on the cell surface by comparing the abundance of enriched proteins versus the control.^145^ To elucidate the surface glycosylation interactome, Sun et al. combined chemical cross-linking with specific enrichment of surface glycoproteins using galactose oxidase and hydrazide chemistry (Figure 10). The method allowed for confident identification of more than 300 proteins interacting with surface glycoproteins.^146^ To identify the sialic acid mediated protein interactions, glycoproteins metabolically labeled with ManNAz were cross-linked with its binding partners using a chemical cross-linker (NHS-cyclooctyne).^147^ Antigen and antibody binding is a critical process on the cell surface that is associated with immune response and cell–cell interactions. A method termed antigen–antibody proximity labeling (AAPL) was designed to map the antigen interacting proteins using the engineered glycosylated antibody that contains Fe(III) for catalyzing the oxidation of the methionine residues on neighboring proteins with the presence of H_2_O_2_.^148^ Glycosylation can also regulate protein aggregation. For example, *O-*GlcNAcylation can prevent the progression of neurodegenerative diseases.^149^ Mechanistically, *O-*GlcNAcylation can inhibit the aggregation of tau and α-synuclein and stabilize them.^150,151^ Moreover, *O-*GlcNAc modifies and increases the ability of small heat shock proteins to block the amyloid formation of both α-synuclein and Aβ(1–42).^152^ It is of great interest to identify more protein substrates whose *O-*GlcNAcylation can prevent their aggregation.

## 4 Quantitative Glycoproteomics for Human Disease Research

### 4.1 Alzheimer’s Disease

The dysregulation of glycosylation is associated with many human diseases, including congenital disorders of glycosylation (CDG), autoimmune disease, cardiac hypertrophy, cancer, diabetes, and neurodegenerative diseases.^62^ Among human organs, *O-*GlcNAcylation is particularly abundant in the brain, and the expression level and activity of OGT and OGA are also high.^153^*O-*GlcNAcylation regulates key cellular processes in the brain, including the neuronal excitability and synaptic release machinery.^154,155^ In neurodegenerative diseases, the level of *O-*GlcNAcylation in the brain significantly decreases, which is related to the loss of memory and learning abilities.^156^ Alteration of *O-*GlcNAcylation on some key proteins (tau, α-synuclein, and Aβ) in neurodegenerative diseases were found, and the increase of *O-*GlcNAcylation on these proteins under the OGA inhibition reduced protein aggregation, prevented the death of neuron cells, and repaired the memory functions.^157−160^ To investigate the change of *O-*GlcNAcylated proteins in Alzheimer’s disease on a global scale, *O-*GlcNAcylated proteins were quantified in the Alzheimer’s disease samples using MS-based proteomics, and *O-*GlcNAcylated proteins with reduced abundances in Alzheimer’s disease were related to synapse, cytoskeleton, neuronal structure, protein degradation, glucose metabolism, and memory.^73,161,162^

**Figure 11:**
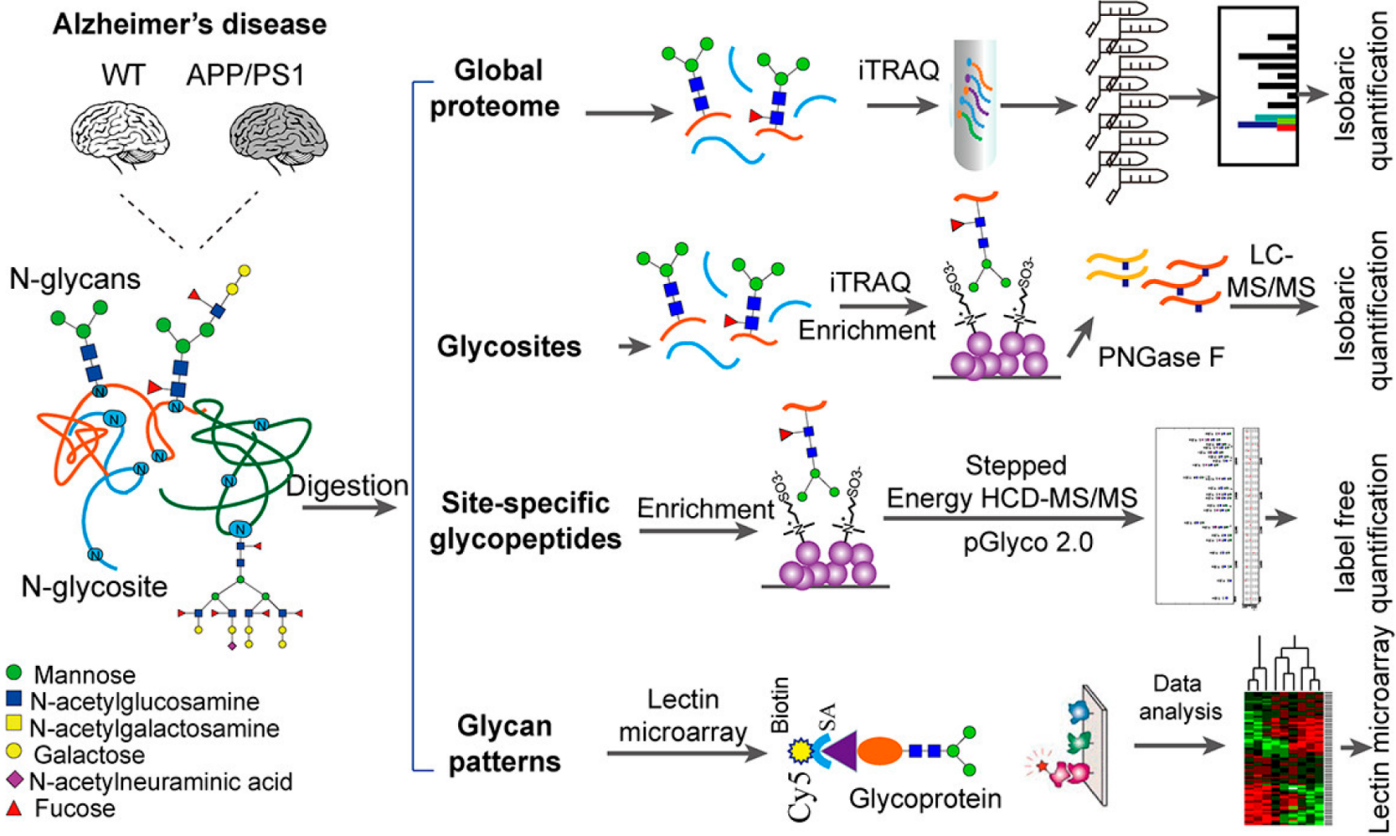
Quantitative analysis of the proteome, *N-*glycosylation sites, site-specific *N-*glycopeptides, and glycan patterns in the mouse brain with Alzheimer’s disease versus control. (Adapted from ref. (164), with the permission from American Chemical Society.)

Besides *O-*GlcNAcylation, other types of glycosylation also play critical roles in the pathology of Alzheimer’s disease.^163^ In a number of studies, *N-*glycosylation in the brains with Alzheimer’s disease and control were systematically compared (Figure 11).^164−167^ Dysregulated *N-*glycosylation in the brain with Alzheimer’s disease was found to affect many processes and pathways, including extracellular matrix, synapse, lysosome, ER dysfunction, cell adhesion, endocytic trafficking, cell signaling dysregulation, and neuroinflammation. The decreased expression of *N-*glycosylation affected the trafficking of some proteins (such as Ncam1) to the membrane. Additionally, fucosylated glycans were reduced in the brain with Alzheimer’s disease. Cerebrospinal fluid is a rich source for the discovery of neurodegenerative disease biomarkers, and glycosylation in the cerebrospinal fluid from Alzheimer’s disease was globally quantified.^168,169^ It was reported that the *N-*glycan structures in the cerebrospinal fluids from the brains with Alzheimer’s disease had the altered level of bisecting GlcNAc and fucosylation. A decreased fucosylation level was also observed for mucin-type *O-*glycosylation in the cerebrospinal fluid from the brain with Alzheimer’s disease.

### 4.2 Cancer

The alteration of protein glycosylation is a common feature of many cancers.^170−172^ A well-known phenomenon is the upregulation of sialic acid on the glycans of the cancer cell surface that give the “don’t eat me” signal to immune cells to evade their clearance.^173,174^ Moreover, the expressions of some truncated mucin-type *O-*glycans are upregulated, including the Tn, STn, TF, and STF antigens.^175^ It is common in cancer cells that the uptake of glucose is dramatically increased with the production of lactate, even in the presence of oxygen (Warburg effect).^176^ For protein *N-*glycosylation, their changes in sera and tissues from various cancer types were quantified.^53,177−180^ Many glycotransferases, lectins, and Siglec-1 were reported to be upregulated in liver cancer.^181^ In prostate cancer, aberrant glycosylation was associated with the change of sialylation, fucosylation, and galactosylation in glycans.^180^ It was revealed that the change of fucosylation level in prostate cancer could be attributed to the abundance changes of enzymes responsible for fucosylation, which were found by measuring the abundance alteration in the whole proteome.^182^

As a nutrient sensor, *O-*GlcNAcylation is pivotal in the progression of cancer, and *O-*GlcNAcylation occurs on a series of transcription factors (such as SP1, c-MYC, p53) that regulate the initiation of cancer-related biological processes.^183,184^*O-*GlcNAcylation changes in breast, colorectal, lung, ovarian cancers were systematically quantified, and some *O-*GlcNAcylated proteins were differentially regulated in each type of cancer.^185−187^ For example, in lung cancer, it was reported that *O-*GlcNAcylation of SAM68 was related to cancer migration and invasiveness.

### 4.3 Diabetes

*O-*GlcNAcylation occurs on many transcription factors and cofactors that regulate gluconeogenesis. It was reported that *O-*GlcNAcylation of PGC-1α prevented the protein from proteasomal degradation, leading to the loss of glucose suppression against gluconeogenesis.^188^ Additionally, *O-*GlcNAcylation negatively regulates insulin signaling by inhibiting multiple components in the signaling pathway.^189,190^ Several glycoproteomic studies were conducted to quantify the changes of glycosylated proteins in diabetes.^129,191^ In diabetic hearts, the abundances of many *O-*GlcNAcylated proteins were altered. As one example, PDHA1 had decreased abundance in diabetic hearts. The *N-*glycosylation analysis in the kidney sample from diabetic mice revealed that there were higher abundances of *N-*glycosylated proteins involved in cell adhesion and cell–matrix composition for mice with diabetes.

### 4.4 Other Diseases

Besides the diseases discussed above, aberrant glycosylation is also related to other diseases. For example, osteoarthritis is a progressive whole-joint disease, and Kashin-Beck disease is a native and chronic deformative osteoarthropathy in contrast to osteoarthritis.^192^ Lyu et al. compared the *N-*glycosylation differences in these two diseases and found some *N-*glycoproteins involved in the pathological processes of both diseases. Moreover, glycosylation may influence the pathological process by affecting the integrity of chondrocytes or cartilage.^193^ Myasthenia gravis is an autoimmune disease that causes weakness and rapid fatigue of muscle.^194^*N-*Glycosylation of IgG-V was elevated in Myasthenia gravis, which may disturb the interactions of IgG with antigens in the disease.^195^ Rheumatoid arthritis is another autoimmune disease that results in inflammation in tissues and joints.^196^ Quantitative glycoproteomics revealed the altered abundances of 29 glycoproteins in Rheumatoid arthritis, and many of them are related to the complement system.^197^ In patients with heart failure, global analysis of glycoproteins revealed the upregulation of disialyl-T *O-*glycosylation and the downregulation of core-fucosylation on *N-*glycans.^198^ Schizophrenia is a serious mental disorder that affects around 2% of people worldwide.^199^ The change of *N-*glycosylation signature was observed in the disease, and the levels of bisecting and sialylated glycans in the cerebrospinal fluid were downregulated.^200^

## 5 Conclusion and Outlook

With the development of MS-based proteomics, now it is possible to globally and site-specifically characterize protein glycosylation. Using quantitative glycoproteomic methods, the abundances of glycoproteins in different samples can be accurately measured, allowing for investigation of the effect of glycosylation on protein properties and functions, and discovery of aberrant glycosylation events in human diseases. In this review, we discuss different quantitative proteomic methods for quantification of glycoproteins. Furthermore, as glycosylation often determines protein properties and functions, we summarize some quantitative glycoproteomic studies for glycoprotein dynamics, stoichiometry, subcellular localization, and interactions with macromolecules. Later on, quantitative studies of glycoprotein alterations in various diseases are discussed because dysregulation of glycosylation is related to multiple diseases. Given the advancement of quantitative glycoproteomics, it will have extensive applications to systematically study the role of protein glycosylation in particular biological processes and diseases, as well as serving as high-throughput screening methods for finding glycoproteins as potential biomarkers.

There are still several issues hindering wider applications of quantitative glycoproteomics. First, modern MS instruments for proteomics are normally very expensive and require significant amounts of resources for maintenance because the mass spectrometers used for proteomic analysis require high speed, resolution, and mass accuracy. Moreover, the isobaric encoded mass tags used for quantitative proteomics, such as TMT-10plex, require an exceptionally high resolution (>45,000) for tandem MS scans. Only very few types of MS analyzers, such as Orbitrap, can fulfill the requirements. This makes it difficult for most laboratories to perform glycoproteomic research. Second, the abundances of many glycoproteins are very low, and enrichment is imperative for global analysis of glycoproteins. However, despite several existing methods, successful enrichment of glycopeptides/glycoproteins in complex biological samples is still not trivial. Even for the same enrichment method, the performance from different laboratories and individuals may vary dramatically due to the technical difficulty of capturing glycoproteins with low abundance. Further development of enrichment methods that are robust, easy to use, and not restrictive to sample sources is urgently needed. Furthermore, the heterogeneity of glycans makes the enrichment even more challenging and the database search more difficult. For example, the size of intact *N-*glycopeptides is very large. In MS2 spectra, the fragments from the glycan and peptide components and the combination of both exist, and they are scattered in a wide *m*/*z* range, complicating peak assignment and annotation. Besides, the optimal dissociation energies for the glycan component, the peptide backbone, and the isobaric mass tag are different. Therefore, the fragmentation of glycopeptides with a certain dissociation energy may not provide sufficient information. Despite these challenges, the technologies for MS-based proteomics are rapidly evolving. In the future, the improvement for MS instrumentation, the development of enrichment methods, and more powerful software for comprehensive analysis of protein glycosylation will aid in further advancing quantitative glycoproteomics-based research for better understanding the properties and functions of glycoproteins and their roles in various diseases.
